# Interpretable machine learning for predicting pathologic complete response in patients treated with chemoradiation therapy for rectal adenocarcinoma

**DOI:** 10.3389/frai.2022.1059033

**Published:** 2022-12-07

**Authors:** Du Wang, Sang Ho Lee, Huaizhi Geng, Haoyu Zhong, John Plastaras, Andrzej Wojcieszynski, Richard Caruana, Ying Xiao

**Affiliations:** ^1^Department of Radiation Oncology, University of Pennsylvania, Philadelphia, PA, United States; ^2^Microsoft Research, Redmond, WA, United States

**Keywords:** interpretable machine learning, radiomics, dosiomics, multi-view input data analysis, rectal cancer, pathologic complete response, clinical image processing

## Abstract

**Purpose:**

Pathologic complete response (pCR) is a critical factor in determining whether patients with rectal cancer (RC) should have surgery after neoadjuvant chemoradiotherapy (nCRT). Currently, a pathologist's histological analysis of surgical specimens is necessary for a reliable assessment of pCR. Machine learning (ML) algorithms have the potential to be a non-invasive way for identifying appropriate candidates for non-operative therapy. However, these ML models' interpretability remains challenging. We propose using explainable boosting machine (EBM) to predict the pCR of RC patients following nCRT.

**Methods:**

A total of 296 features were extracted, including clinical parameters (CPs), dose-volume histogram (DVH) parameters from gross tumor volume (GTV) and organs-at-risk, and radiomics (R) and dosiomics (D) features from GTV. R and D features were subcategorized into shape (S), first-order (L1), second-order (L2), and higher-order (L3) local texture features. Multi-view analysis was employed to determine the best set of input feature categories. Boruta was used to select all-relevant features for each input dataset. ML models were trained on 180 cases from our institution, with 37 cases from RTOG 0822 clinical trial serving as the independent dataset for model validation. The performance of EBM in predicting pCR on the test dataset was evaluated using ROC AUC and compared with that of three state-of-the-art black-box models: extreme gradient boosting (XGB), random forest (RF) and support vector machine (SVM). The predictions of all black-box models were interpreted using Shapley additive explanations.

**Results:**

The best input feature categories were CP+DVH+S+R_L1+R_L2 for all models, from which Boruta-selected features enabled the EBM, XGB, RF, and SVM models to attain the AUCs of 0.820, 0.828, 0.828, and 0.774, respectively. Although EBM did not achieve the best performance, it provided the best capability for identifying critical turning points in response scores at distinct feature values, revealing that the bladder with maximum dose >50 Gy, and the tumor with maximum2DDiameterColumn >80 mm, elongation <0.55, leastAxisLength >50 mm and lower variance of CT intensities were associated with unfavorable outcomes.

**Conclusions:**

EBM has the potential to enhance the physician's ability to evaluate an ML-based prediction of pCR and has implications for selecting patients for a “watchful waiting” strategy to RC therapy.

## Introduction

More than 40,000 patients in the United States are diagnosed with rectal cancer (RC) annually, with 70% of them in advanced stages. Preoperative neoadjuvant chemoradiation therapy (nCRT) can reduce local recurrence rates and is considered standard treatment for locally advanced rectal cancer (LARC) with subsequent total mesorectal excision. Several recent studies and clinical trials have demonstrated that patients with a pathological complete response (pCR) after nCRT have the best tumor control and survival rates and can benefit from “watch-and-wait” approaches (Maas et al., 2010; Colorectal Cancer: Statistics, 2012). This organ-preservation strategy can protect patients from the morbidity, mortality, and functional disorders caused by radical surgery (Habr-Gama et al., 2019; El Sissy et al., 2020; Fernandez et al., 2020; São Julião et al., 2020; Sun et al., 2020). Therefore, identifying crucial predictors of pCR is essential for assisting clinicians in selecting the most effective treatment for individual patients.

Radiomics (R), an emerging field of translational research, aims to extract quantitative image features, which can be categorized as shape, first-order, texture, and filter-based features. A number of studies have shown that R features can distinguish between tumor stages (Ganeshan et al., 2010; Dong et al., 2013), are associated with cancer genetics, and are helpful for outcome prediction (Liu et al., 2017; He et al., 2018; Wang et al., 2019). Dosiomics (D) has been proposed as a method to extract spatial attributes from a 3D dose distribution in contrast to conventional point-wise dose-volume histogram (DVH) parameters, and it can add value for a more thorough assessment of dose toxicity (Rossi et al., 2018; Liang et al., 2019; Lee et al., 2020).

Several recent studies have demonstrated that machine learning (ML) models that incorporate R and D features are highly effective in predicting radiotherapy (RT) outcomes (He et al., 2018; Giraud et al., 2019; Chen et al., 2022). However, these approaches frequently lack sufficient interpretability, which limits their use in clinical decision-making. In this study, we implemented an ML glass-box model called the explainable boosting machine (EBM) to predict pCR and attain interpretability while still providing optimal performance. The EBM, which is a tree-based, cyclic gradient boosting generalized additive model designed to provide both intelligibility and high accuracy, has been used to find significant and unexpected impacts in healthcare data (Lou et al., 2013; Caruana et al., 2015; Lundberg and Lee, 2017).

Due to the high dimension of R and D features, the over-fitting problem may arise with a relatively small training dataset, which may have an impact on both the feature selection procedure and the prediction performance. To date, most R studies have used a single-view concatenated input, which just aggregates all available features, and thus have typically shown limited or suboptimal model performance (Lao et al., 2017; Wang et al., 2019). Recently, Lee et al. showed that the use of a multi-view R and D analysis strategy can significantly improve the performance of predicting acute-phase weight loss in lung cancer patients undergoing radiotherapy (Lee et al., 2020). In this strategy, R and D features are subcategorized into shape (S), first-order (L1), second-order (L2), and higher-order (L3) local texture features. Multi-view input feature sets are constructed using all possible subcategory combinations. We postulate that the multi-view R and D analytics can help us find the optimal set of features for predicting radiotherapy outcomes in RC while improving the predictability and interpretability of an ML model.

In this study, we evaluated the performance of the EBM model in predicting pCR of patients with LARC treated with nCRT in comparison to several state-of-the-art black-box ML models based on multi-view input data analysis with clinical parameters (CPs), DVH parameters, R and D features. In addition to the interpretation performed using the EBM model, the outputs of all the black-box ML models were interpreted using Shapley additive explanation (SHAP) values, allowing for comparison of the interpretation of the various ML models.

## Materials and methods

### Dataset

This study utilized two independent datasets and was approved by the institutional review board (No. 831721) at the University of Pennsylvania. Patients diagnosed with RC and who received preoperative chemoradiation therapy at the University of Pennsylvania between 2008 and 2016 were included as a training cohort. An independent testing cohort included patients with LARC who were registered in a multi-institutional clinical trial, RTOG 0822 (a phase II evaluation of preoperative chemoradiotherapy utilizing intensity-modulated radiation therapy in combination with capecitabine and oxaliplatin for patients with LARC). Patients enrolled in the RTOG 0822 were treated between 2008 and 2009 and followed up until 2015. We enrolled a total of 180 patients from our institution and 37 patients from the RTOG 0822 clinical trial. Patients in both cohorts received nCRT followed by surgical resection. The patient's pCR status was confirmed by histopathologic analysis of the surgical specimens obtained. Patients whose information was missed were excluded. The DICOM files of eligible patients were collected and uploaded to MIM (MIM Software Inc., Cleveland, OH, USA) for initial review. These DICOM files included pre-treatment CT images, radiotherapy plan, RT structure, and RT dose. Gross tumor volume (GTV) was manually delineated, and experienced radiologists evaluated its appropriateness and corrected it if necessary.

### Pathologic complete response

To determine the patient's pCR status, we evaluated the pathologist-reported tumor stage based on specimens obtained during the surgery, i.e., the total mesorectal excision. The pCR was defined as complete tumor regression with no viable cancer cells in both primary tumors and regional lymph nodes found upon histologic examination of the surgical specimen by a pathologist.

### Dose-volume histogram (DVH) parameters

The RTOG 0822 protocol compliance criteria imposed a total of 14 dose constraint points on four structures: D98%[Gy], D10%[Gy], D5%[Gy], and Dmax[Gy] for the planning target volume (PTV); D180cc[Gy], D100cc[Gy], D65cc[Gy], and Dmax[Gy] for the small bowel; D40%[Gy], D25%[Gy], and Dmax[Gy] for the femoral heads, and D40%[Gy], D15%[Gy], and Dmax[Gy] for the bladder. To avoid inconsistencies in the target definition, GTV was used instead of PTV for some patients treated with procedures and prescription doses other than the RTOG 0822 criteria. In addition to the dose-volume points for organs-at-risk (OARs) required by the RTOG 0822 protocol, we added extra points ranging from D5%[Gy] to D95%[Gy], in 5% steps to the GTV, femoral heads, and bladder, and from D10cc[Gy] to D180cc[Gy], in 10cc steps for the small bowel. The DVH metrics were obtained from the DVH reviewer via the MIM for each patient, and a MATLAB-based dose/volume point statistics (DPS) tool was used to extract the DVH parameters at the designated dose-volume points (Gong et al., 2016).

### Radiomics (R) and dosiomics (D) features

To investigate the spatial distribution of gray values in an image, R and D features on CT images and a 3D dose map were extracted from the GTV, respectively. A total of 107 R features from the pre-treatment CT images and 93 D features from the 3D dose map were extracted using an open-source Python library called Pyradiomics (ver. 3.0) (van Griethuysen et al., 2017). The extracted R and D features were summarized in Supplementary Table 1. The air bubble in GTV was removed from the original CT images for computing R and D features by excluding CT values below −200. The 3D dose map was produced by resampling dose grids with b-spline interpolation to have the same spatial resolution as its corresponding CT images. The R and D features included 3D shape (14 features for R only), first-order statistics (18 features), and texture features computed from gray level co-occurrence matrix (GLCM, 24 features), gray level dependence matrix (GLDM, 14 features), gray level run length matrix (GLRLM, 16 features), gray level size zone matrix (GLSZM, 16 features), and neighboring gray tone difference matrix (NGTDM, 5 features). Only original images without any filtering were used to extract both R and D features for the sake of intuitive model interpretation. These R and D features were extracted from the region of the images enclosed by a 3D bounding box that was cropped around the GTV with voxel padding (10 voxels). Bin widths were set to 25 HU and 25 cGy for the discretization of R and D features, respectively.

### Multi-view input feature sets

The R and D texture features were classified into three categories: L1 (first-order statistics), L2 (second-order local texture statistics: GLCM and GLDM), and L3 (higher-order local texture statistics: GLRLM, GLSZM, and NGTDM), depending on the number of pixels defining the local feature. The L1 statistics describe the properties of individual pixel values, whereas the L2 and L3 statistics estimate the properties of two or more-pixel values co-occurring at particular locations. Multi-view input feature sets were constructed concatenating all CPs and DVH parameters with each of all possible combinations of the R and D subcategories (*n* = 64). We defined one of the multi-view input feature sets as the single-view input that concatenated all the features, i.e., CP+DVH+Rall+Dall, where Rall and Dall denote S+R_L1+R_L2+R_L3 and D_L1+D_L2+D_L3, respectively. For the sake of simplicity in the multi-view input data analysis, 3D shape features from the S category were merged with the L1 category of R features rather than being used as a separate feature category.

### Feature selection

For each training set of multi-view and single-view input features, Boruta algorithm was used to remove any redundant features and screen for highly associated features with patient outcomes. The Boruta functions as a wrapper algorithm for the random forest (RF). This algorithm generates shuffled copies (shadow features) of all input features before training the RF classifier with the extended dataset. The Z-score of each feature in the extended dataset is then calculated. The Z-score measures the number of standard deviations a data point is from the population mean. During each iteration, features with a higher Z-score than their shadow features are marked as essential; otherwise, they are removed from the features. The iteration ends when all input features are confirmed and rejected, or when the user preset runs are reached. The Boruta algorithm was implemented using Python package Boruta (ver. 0.3) (Kursa and Rudnicki, 2010) with a maximum iteration of 1,000 and a significance level of *p* < 0.05.

### Classifiers

Features selected by the Boruta were used to train classification models for pCR prediction. Four classifiers were used in this study, including the EBM and three black-box ML algorithms: extreme gradient boosting (XGB), RF, and support vector machine (SVM) with a radial basis function kernel. The EBM is a glass-box model with direct interpretability and high prediction performance comparable to state-of-the-art ML methods. In this study, the EBM models were constructed using Python package interpret (ver. 0.2.6), developed by Microsoft (Nori et al., 2019). The XGB is a widely employed and very effective ML method that belongs to boosting ensemble learning, which provides a parallel tree boosting and is highly efficient, flexible, and portable. Python package xgboost (ver. 1.5.2) was used to build our XGB models (Chen and Guestrin, 2016). The RF is a decision tree that extends the bagging and ensemble learning method. The RF algorithm generates many decision trees with each of them using bagging and feature randomness. The RF is simple to use and provides a higher level of prediction accuracy in many tasks. The RF models were built based on RandomForestClassifier (Breiman, 2001) from Python package sklearn (ver. 0.24.2). The SVM is a supervised ML algorithm that aims to find a hyperplane with the optimal boundary to separate different categories of samples. Support vector machine provides good generalization ability and is commonly applied in solving small-sample, non-linear, and high-dimensional space recognition. To build the SVM models, we used the support vector classification module (Suykens and Vandewalle, 1999) from the Python library sklearn (ver. 0.24.2).

### Performance evaluation

Before training the models, the training data were standardized to have zero mean and unit variance and the test data were scaled with these statistics of the training data. In addition, the synthetic minority oversampling technique (SMOTE) was used to address a problem introduced by class imbalance. The SMOTE is an oversampling technique where the synthetic samples are generated for the minority class (Chawla et al., 2002). This approach uses interpolation between the positive instances that are close to one another to create new instances, hence addressing the overfitting issue caused by random oversampling. Hyperparameters were optimized using five-fold cross-validation (CV) in grid search for all of the ML models investigated. The five-fold CV is performed with four folds for training and the remaining fold for validation, which is repeated until every fold is used for validation. The final step in training is to average all CV performances in order to represent the performance for each possible combination of candidate hyperparameters as discrete grid points and choose the optimal set of hyperparameters that can yield the best performance. The performance of all ML models trained for predicting pCR was evaluated in the external test dataset using the area under the receiver operating characteristic curve (ROC AUC) and the harmonic mean of precision and recall (F1 score). The DeLong test was used to compare the ROC curves for each model generated from its optimized multi-view input feature set with that generated from the single-view input feature set (DeLong et al., 1988).

### Model interpretation

**Figure 1:**
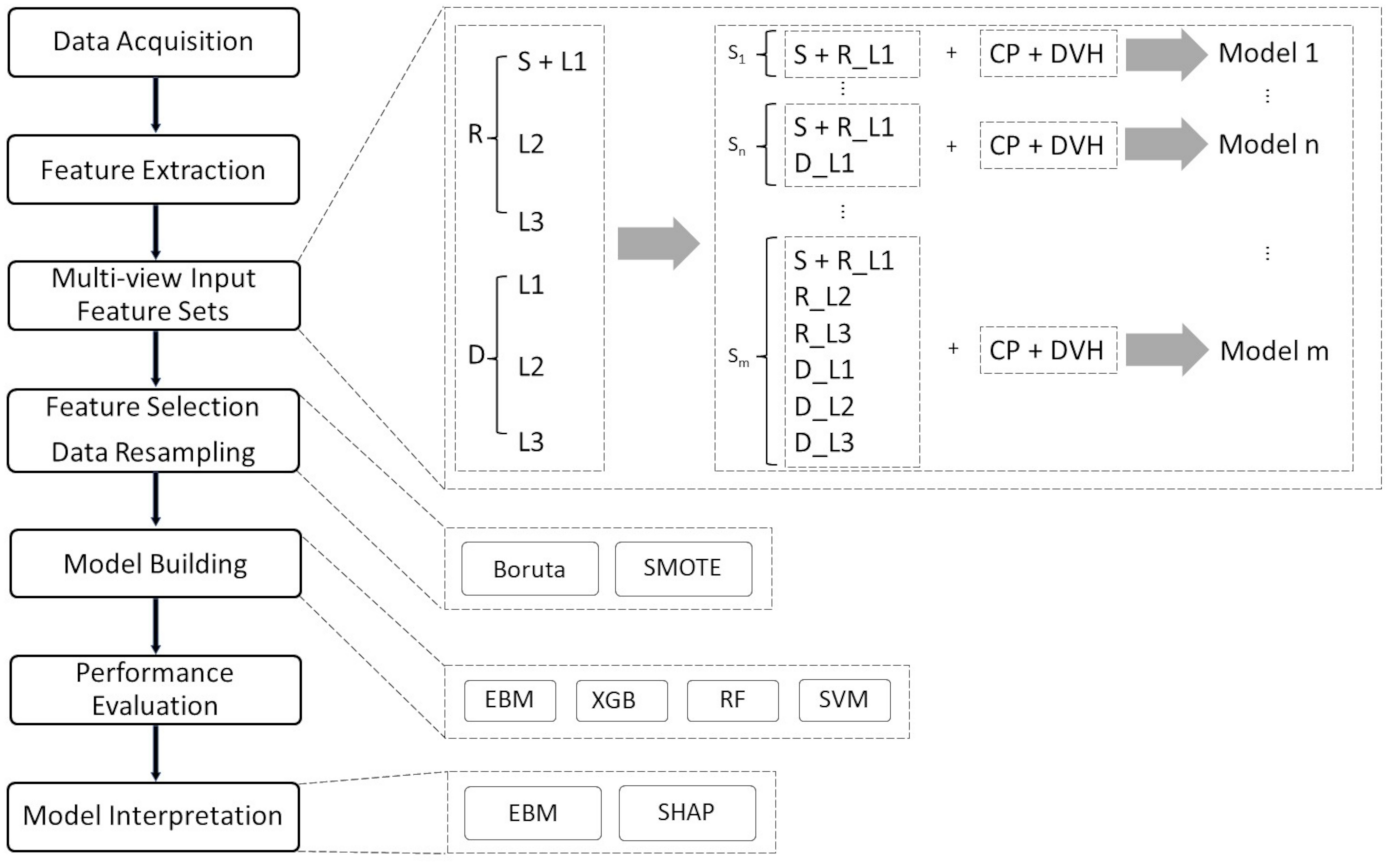
Interpretable ML pipeline for pCR prediction. The entire procedure consists of eight distinct phases: data collection and cleaning, feature extraction, multi-view input feature categorization, feature selection, data resampling, model building, performance evaluation, and model interpretation. In the data cleaning session, cases with missing data and incomplete treatment (treatment interruptions) are removed from the cohort.

Shapley additive explanations, a game theoretic approach, were used to interpret the output of the black-box ML models (Lundberg and Lee, 2017). Overall feature importance was calculated in the training dataset using the mean absolute values of the EBM scores and the mean absolute SHAP values for the black-box ML models. To visualize how each ML model uses a specific feature in the classification task, EBM shape and SHAP-based partial dependence functions were plotted for each feature. The EBM shape function is the gradient-boosted ensembles of trees where each tree deals with a single feature, while the SHAP-based partial dependence function represents the marginal effect of a single feature on the outcomes predicted by a black-box ML model. In this study, SHAP was implemented in the Python package shap (ver. 0.39.0). Figure 1 displays our interpretable ML pipeline, which includes the above-mentioned multi-view input, feature selection, model building, and model interpretation phases.

## Results

### Patient characteristics

Table 1 lists the clinical data and patient demographics for each cohort. Following pre-screening, we included 180 patients in our training cohort, of whom 42 (23.3%) achieved pCR, and 37 patients in the test cohort, of whom 6 (16.2%) obtained pCR. Each patient had 11 CPs collected: gender, race, ethnicity, age, clinical stage (AJCC 7th edition), treatment modality, RT prescription dose, surgical procedure, time from preoperative therapy to surgery, adjuvant chemotherapy, and pCR. There was no statistical difference in gender, race, prescription dose, surgery procedure, and pCR status between the two cohorts. Except for pCR, all CPs were used to train the classifiers.

**Table 1:** Clinical and demographic characteristics of enrolled patients.

**Cohort**	**Training**	**Testing**	***p*-Value**
Number of patients	180	37	
**Clinical diagnosis**			
Gender			0.62
Male	110 (61.1%)	21 (56.8%)	
Female	70 (38.9%)	16 (43.2%)	
**Race**			
White	141 (78.3%)	34 (91.9%)	0.11
Black	25 (13.9%)	3 (8.1%)	
Other	14 (7.8%)	0 (0%141)	
Age			< 0.0001
Mean **±** SD	62.7 ± 11.8	53.8 ± 10.2	
Ethnic			< 0.01
Hispanic or Latino	4 (2.2%)	4 (10.8%)	
Not Hispanic or Latino	175 (97.2%)	31 (83.8%)	
Unknown	1 (0.6%)	2 (5.4%)	
Clinical T stage			0.03
I	0 (0%)	0 (0%)	
II	26 (14.4%)	0 (0%)	
III	140 (77.8%)	32 (86.5%)	
IV	14 (7.8%)	5 (13.5)	
Clinical N stage			0.11
0	50 (27.8%)	16 (43.2%)	
I	115 (63.9%)	16 (43.2%)	
II	14 (7.8%)	5 (13.5%)	
III	1 (0.5%)	0 (0%)	
Clinical M stage			0.13
0	166 (92.2%)	37 (100.0%)	
I	14 (7.8%)	0 (0%)	
**Treatment**			
Prescription dose			0.37
Standard (50.4 Gy)	150 (83.3%)	33 (89.2%)	
Non-standard	30 (16.7%)	4 (10.8%)	
Treatment modality			< 0.01
Photon	144 (80%)	37 (100.0%)	
Proton	36 (20%)	0 (0%)	
Surgery procedure			0.11
LAR	117 (65%)	16 (43.2%)	
APR	57 (31.7%)	9 (24.3%)	
Other	6 (3.3%)	12 (32.4%)	
Neoadjuvant chemotherapy			< 0.0001
5FU	84 (46.7%)	0 (0%)	
Capecitabine	61 (33.9%)	0 (0%)	
5-FU and oxaliplatin	31 (17.2%)	0 (0%)	
Capecitabine and Oxaliplatin	4 (2.2%)	37 (100.0%)	
Interval from preoperative therapy to surgery (Days)			< 0.0001
Mean **±** SD	62.7 ± 11.8	53.8 ± 10.2	
pCR			0.34
Yes	42 (23.3%)	6 (16.2%)	
No	138 (76.7%)	31 (83.8%)	

The Student's *t*-test was used to compare the continuous variables between the training and testing cohorts, whereas the Chi-square test or Fisher exact test was implemented for categorical variables. LAR, low anterior resection; APR, abdominal perineal resection.

### Model performance

The performance of the top 10 multi-view and single-view inputs for the EBM, XGB, RF, and SVM classifiers is detailed in Supplementary Tables 2–5, respectively. Table 2 summarizes the performance of only the best multi-view and single-view inputs for the EBM, RF, SVM, and XGB classifiers in pCR prediction, the RF classifier yielded the highest testing performance [Matthews correlation coefficient = 0.403, F1 score = 0.5, accuracy = 0.838, and AUC = 0.828 (95% CI 0.694–0.941)] and was closely followed by the EBM classifier [Matthews correlation coefficient = 0.356, F1 score = 0.471, accuracy = 0.757, and AUC = 0.820 (95% CI 0.680–0.929)]. The XGB classifier showed the lowest Matthews correlation coefficient of 0.303 and F1 score of 0.429, and the SVM classifier provided the lowest test accuracy of 0.676 and AUC of 0.774 (95% CI 0.621–0.906) in comparison to other models. The training performances of all models were comparable between the single-view and optimized multi-view input feature sets, while the testing performance was significantly higher when using the optimized multi-view input feature set.

**Table 2:** Comparison of performances in pCR prediction for four different models built with features selected from Boruta using the best multi-view input (CP+DVH+S+R_L1+R_L2), and the single-view input concatenated with full features (CP+DVH+R_all_+D_all_).

**Input feature set**	**Model**	**Dataset**	**Sensitivity**	**Specificity**	**Matthews**	**F1 score**	**Accuracy**	**AUC 95% (CI)**
Best Multi-view	EBM	Training	0.841	0.667	0.515	0.773	0.754	0.762 (0.760, 0.764)
		Testing	0.667	0.774	0.356	0.471	0.757	0.820 (0.680, 0.929)
	RF	Training	1	0.993	0.993	0.996	0.996	0.821 (0.820, 0.823)
		Testing	0.5	0.903	0.403	0.5	0.838	0.828^*^ (0.694, 0.941)
	SVM	Training	0.913	0.645	0.579	0.805	0.779	0.725 (0.707, 0.742)
		Testing	0.833	0.645	0.356	0.455	0.676	0.774^*^ (0.621, 0.906)
	XGB	Training	0.986	0.935	0.921	0.961	0.96	0.820 (0.819, 0.821)
		Testing	0.5	0.839	0.303	0.429	0.784	0.828^*^ (0.694, 0.941)
Single-view	EBM	Training	0.877	0.754	0.635	0.826	0.815	0.772 (0.770, 0.774)
		Testing	0.333	0.774	0.092	0.267	0.703	0.624 (0.401, 0.817)
	RF	Training	1	1	1	1	1	0.858 (0.856, 0.861)
		Testing	0	0.903	−0.131	N/A	0.757	0.575 (0.381, 0.758)
	SVM	Training	0.957	0.775	0.744	0.877	0.866	0.795 (0.776, 0.813)
		Testing	0.5	0.774	0.228	0.375	0.73	0.522 (0.274, 0.788)
	XGB	Training	1	1	1	1	1	0.814 (0.810, 0.819)
		Testing	0	0.839	−0.174	N/A	0.703	0.484 (0.305, 0.657)

The sensitivity, specificity, Matthews correlation coefficient, F1 score, accuracy, and AUC values for both training and testing are reported. The DeLong test are employed for comparing the testing AUCs for each model between the best multi-view input and the single-view input. The asterisks (*) indicate a statistically significant difference in testing AUC between the best multi-view input and the single-view input.

### Selected features

Boruta selected five features from the optimized multi-view input feature set (CP+DVH+S+R_L1+R_L2): GTV_elongation (the square root of the ratio between the two largest principal components, where 1 indicates a circular tumor and 0 indicates a maximally elongated tumor), GTV_maximum2DDiameterColumn (the largest pairwise Euclidean distance between tumor surface mesh vertices in the row-slice plane), GTV_R_variance (the mean of the squared distances of each intensity value from the mean value, where a larger value is associated with higher heterogeneity of the tumor), bladder_Dmax (the maximum dose received by bladder), and GTV_leastAxisLength (the smallest axis length on the ROI-enclosed ellipsoid).

For single-view input, Boruta returned nine features including GTV_elongation, GTV_maximum2DDiameterColumn, GTV_R_variance, GTV_leastAxisLength, GTV_R_gldm_SmallDependenceLowGrayLevelEmphasis, GTV_R_glszm_GrayLevelNonUniformity, GTV_D_glrlm_LongRunEmphasis, GTV_D_glszm_SizeZoneNonUniformity, and GTV_D_ngtdm_Complexity.

### Feature importance

**Figure 2:**
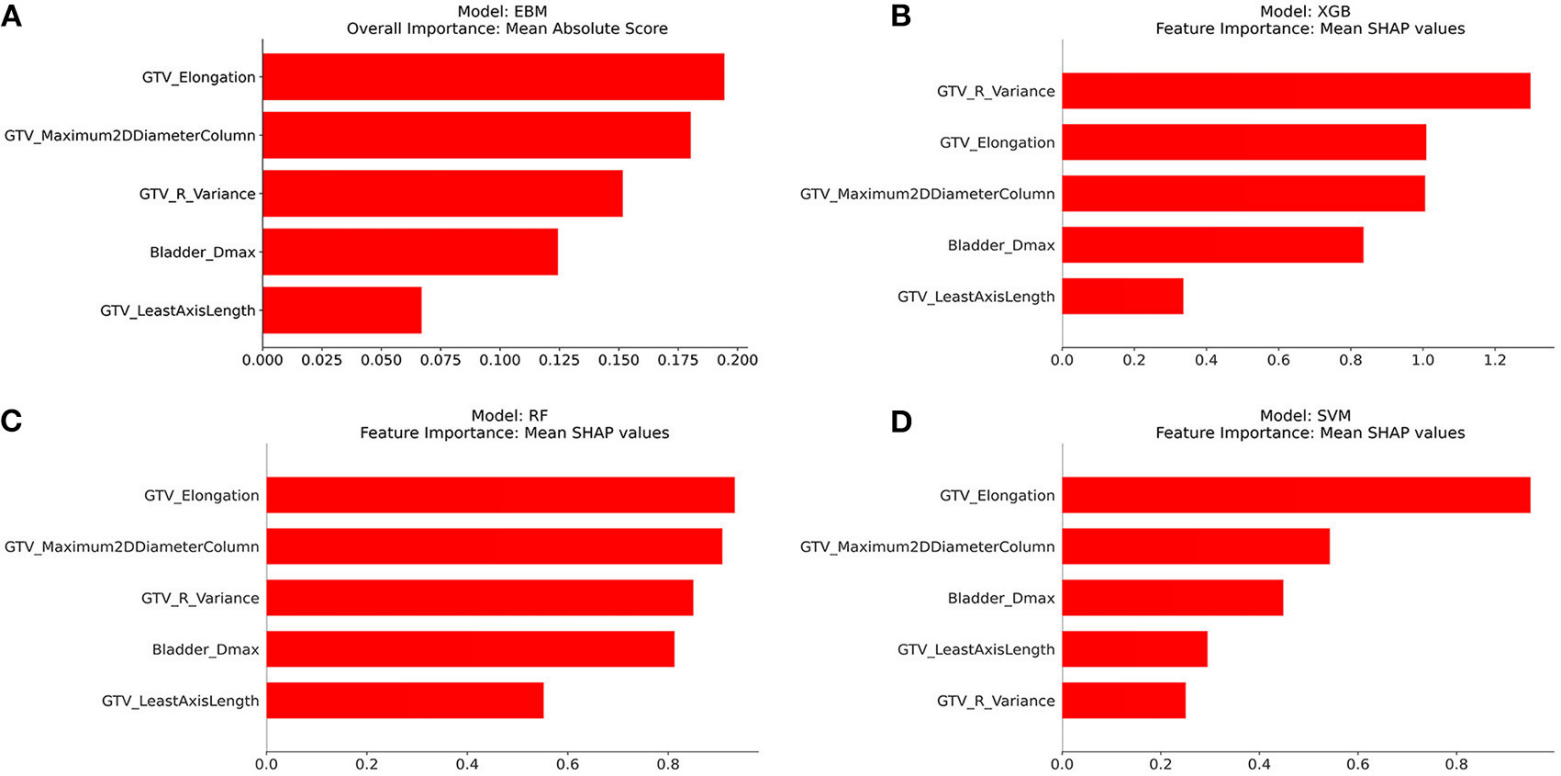
Overall importance of five features selected using Boruta from the best multi-view input. **(A)** EBM, **(B)** XGB, **(C)** RF, and **(D)** SVM.

Figures 2A–D show the overall importance of the five selected features used to build the EBM, XGB, RF, and SVM models, respectively. GTV_elongation was generally found to be the most important feature along with GTV_maximum2DDiameterColumn, followed by bladder_Dmax and GTV_leastAxisLength. The EBM and RF models ranked GTV_R_variance as the third most important feature, whilst the XGB and SVM models ranked it as the most and least important feature, respectively.

### Model interpretation

**Figure 3:**
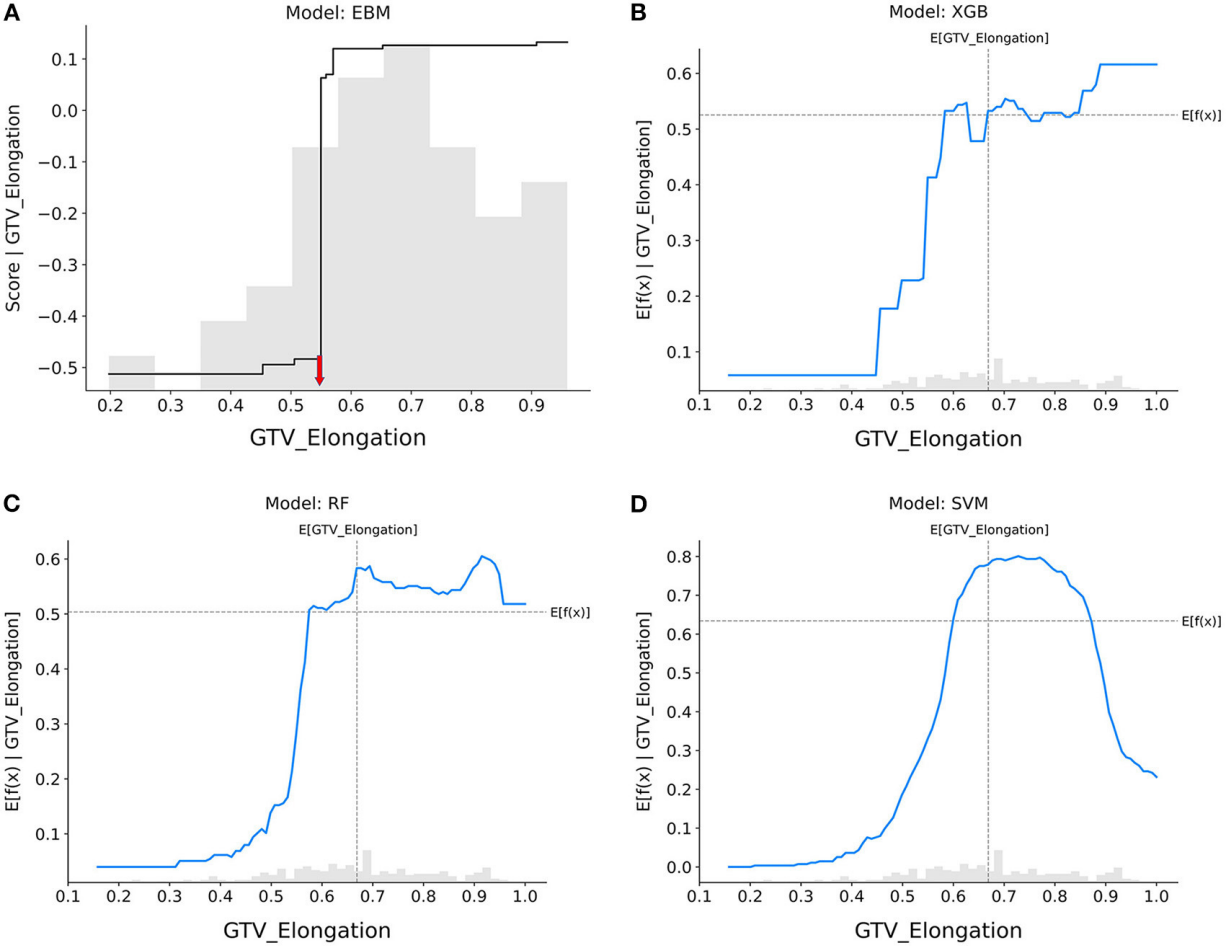
Response score plots for GTV_elongation. **(A)** EBM shape function and SHAP partial dependence functions for the **(B)** XGB, **(C)** RF, and **(D)** SVM models.

**Figure 4:**
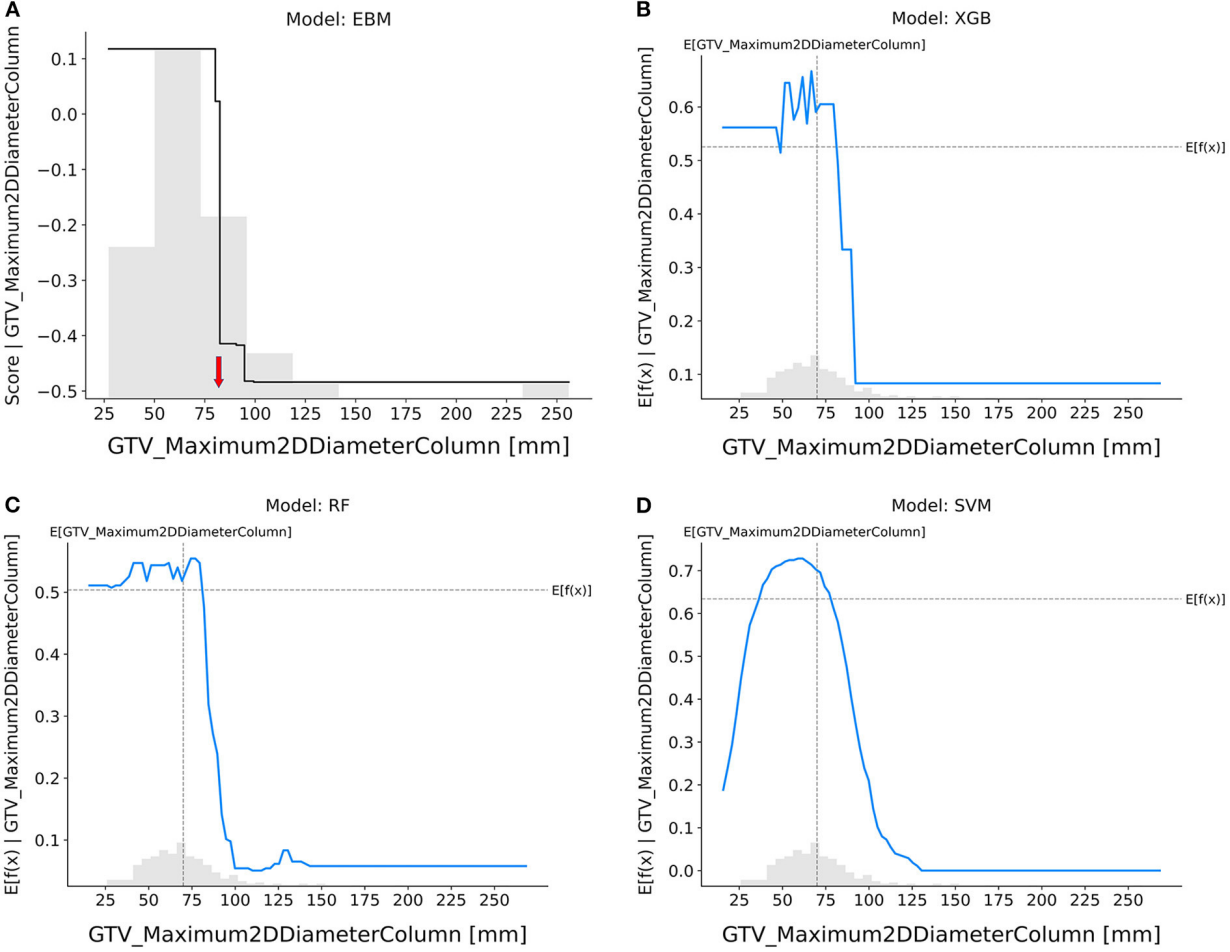
Response score plot for GTV_maximum2DDiameterColumn. **(A)** EBM shape function and SHAP partial dependence functions for the **(B)** XGB, **(C)** RF, and **(D)** SVM models.

**Figure 5:**
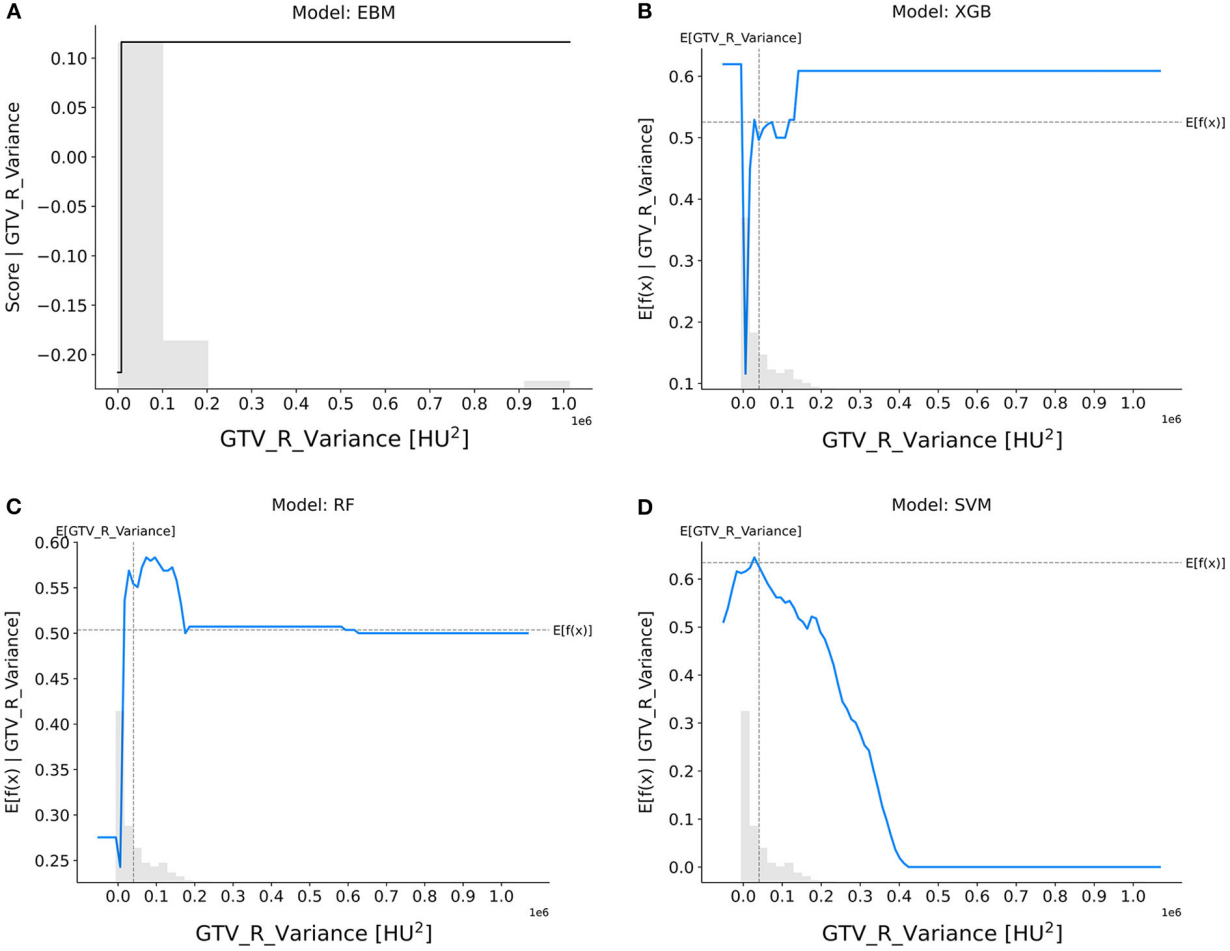
Response score plots for GTV_R_variance. **(A)** EBM shape function and SHAP partial dependence functions for the **(B)** XGB, **(C)** RF, and **(D)** SVM models.

**Figure 6:**
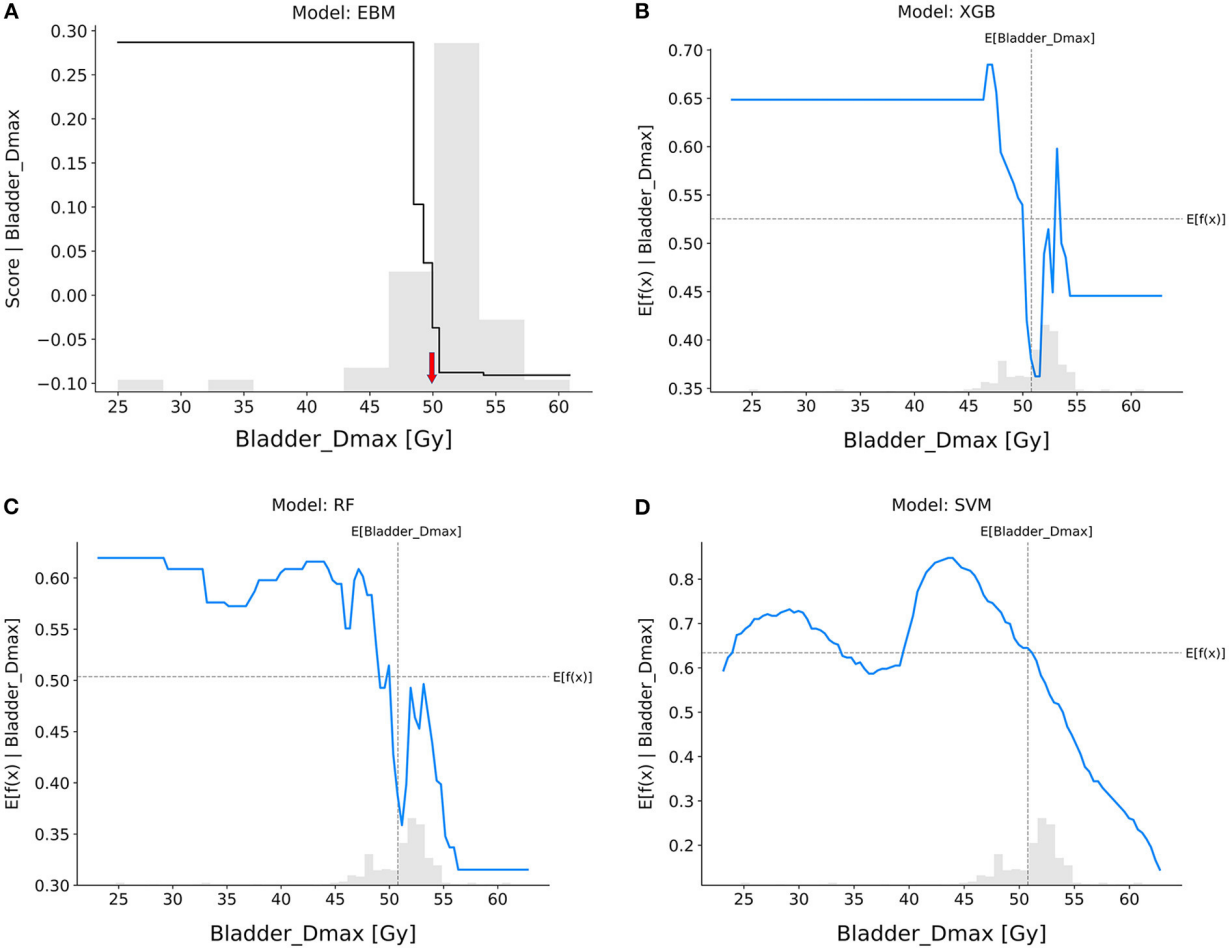
Response score plots for bladder_Dmax. **(A)** EBM shape function and SHAP partial dependence functions for the **(B)** XGB, **(C)** RF, and **(D)** SVM models.

**Figure 7:**
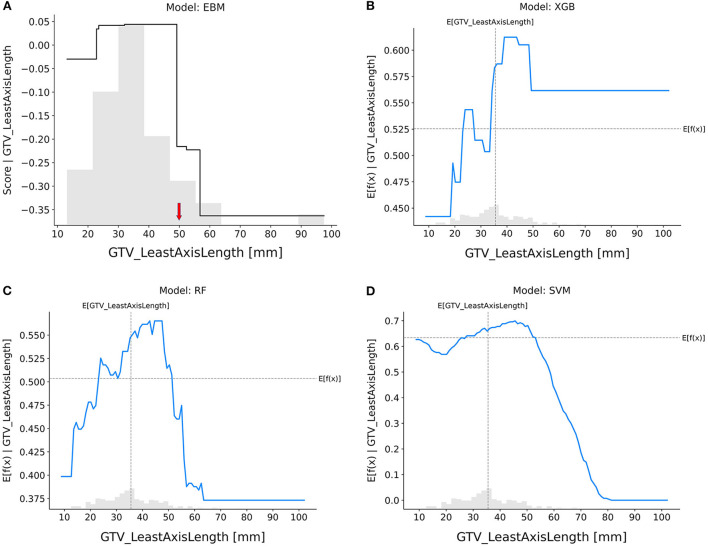
Response score plots for GTV_leastAxisLength. **(A)** EBM shape function and SHAP partial dependence functions for the **(B)** XGB, **(C)** RF, and **(D)** SVM models.

Figures 3–**7** illustrate the contributions of five selected features to the predictions made by each model, respectively. The horizontal axis represents the actual value of the features, while the vertical axis represents the response score. A higher response score indicates a greater contribution to the positive class (pCR), whereas a lower response score indicates a lower likelihood of achieving the pCR. The shadow bar chart is the histogram of the features, which shows the distribution of feature values.

GTV_elongation (Figure 3): When the value of elongation reached between 0.4 and 0.6, the response scores of all models showed a significant increase, indicating that patients with tumors presenting regular circles are more likely to achieve pCR. Only the SVM model showed that increased elongation (>0.8) led to a decrease in the probability that tumors would be eradicated by nCRT.

GTV_maximum2DDiameterColumn (Figure 4): The probability of achieving pCR decreased sharply in the EBM, RF, and XGB models if the tumor's maximal diameter on a longitudinal section was higher than 80 mm, but it did so when tumors were smaller in the SVM model as well.

GTV_R_variance (Figure 5): The EBM, XGB, and RF models suggested that tumors with smaller variance in CT intensities were more resistant to nCRT, whereas the SVM model showed the opposite trend.

Bladder_Dmax (Figure 6): The trend on Bladder Dmax was generally consistent across all models, with patients having a lower chance of achieving pCR when the maximum dose received by the bladder was higher than 50 Gy.

GTV_leastAxisLength (Figure 7): In the EBM, RF, and SVM models, tumors with longer leastAxisLength values showed higher resistance to nCRT. The XGB and RF models exhibited divergent trends, indicating that there is still a risk when the leastAxisLength of GTV is short. The overall trend of response scores in the EBM shape and SHAP-based partial dependence functions for each feature was roughly consistent across different models, indicating that patients with larger tumor sizes, more elongated tumor shapes, and lower variance of CT intensities were less likely to achieve pCR after nCRT.

The results of a case study can be found in the Supplementary material. Supplementary Figure 1 shows a radar chart that compares the values of five selected features between a pCR patient and a non-pCR patient from the testing cohort. Supplementary Figures 2, 3 illustrate the local explanation plots of the EBM and RF models for these patients, which both demonstrate reasonable prediction outcomes.

## Discussion

A large number of studies and clinical trials have shown that pCR is significantly related to local control, distant metastasis, and disease-free survival. This has also promoted the organ preservation program as a very attractive alternative. Therefore, predicting the risk of an individual in advance using non-invasive methods is essential for clinicians to prescribe a personalized treatment plan. Although some recent radiomics research has reported relatively satisfactory model performance in pCR prediction, the use of radiomics-based ML models in clinical settings is still questionable due to their limited interpretability (Nie et al., 2016; Cui et al., 2019; Li et al., 2019). In our study, we carefully constructed our feature selection pipeline to eliminate feature redundancy, and an independent multi-institutional test dataset was used to demonstrate generalizability in the model's performance. Furthermore, this study demonstrated that EBM generates an accurate and reliable model for pCR prediction and interpretation.

The EBM model itself provided transparency regarding predictions, and the EBM shape function informed the response score for each feature. In particular, detecting significant turning points in the response score for each feature was made easier by the monotonicity of the EBM shape function. By contrast, the black-box models (XGB, RF, and SVM) could not be fully transparent by the *post-hoc* explanations using SHAP, because uncertainty existed around how much the local linear approximation of the black-box model (extrapolated on a global scale using SHAP) actually revealed about the original model. This lack of knowledge turned SHAP into a black box with similar complexity as the black box model, when SHAP tried to approximate the behavior of the black-box model by extracting relationships between the feature values and the predictions.

In this study, we demonstrated the value of R and D features in predicting pCR in patients with RC treated with nCRT. In particular, selected R features showed a trend of relatively high feature importance, and the response score function also clearly reflected their strong association with the outcome. Our findings indicated that patients with less elongation in tumor shape, smaller tumors in longitudinal dimension, higher variance in intratumoral CT intensity values, and lower maximum dose to the bladder were at lower risk and could benefit from the watch-and-wait strategy for improved quality of life (Diaz et al., 2012; Jamal-Hanjani et al., 2015; Frame et al., 2017; Dagogo-Jack and Shaw, 2018). The EBM provided a reliable interpretation of the clinical importance of such features.

We also looked into EBM models with multi-view feature inputs. Hundreds of features from the medical image and dose map can be included in R and D features as needed, which could result in a larger number of features being used than the number of patients included in the study and may cause the overfitting issue. Our results demonstrated that the multi-view input data analysis improves feature selection outcomes and performance when compared to single-view concatenated input; however, more research is needed to determine whether our findings consistently will work with alternative feature selection strategies while optimizing the multi-view input data (Kursa and Rudnicki, 2010; De Jay et al., 2013).

Despite the encouraging results, several limitations to this study should also be noted. First, our sample size was relatively small in both training and test cohorts. This made feature selection challenging, and thus we reduced the Z-score threshold for shadow features in Boruta. Second, there were insufficient positive cases with considerable class imbalance in the independent multicenter test dataset, which hindered the model's prediction performance. Third, in comparison to other models, the SVM model showed an inconsistent trend for R features. The SVM may incorrectly classify the data when constructing the decision boundary due to a lack of adequate positive data, which would lower the model's accuracy. Fourth, although we attempted to optimize the input feature set using multi-view data analysis, we did not take into account the relationships between the various feature categories. A further adjustment of the degree of association between different feature categories using a data integration technique may allow for the finding of a more optimal input feature set (Lee et al., 2021). Finally, some recent studies have proved the benefits of MRI scans for collecting advanced textural characteristics and for outcome prediction (Aker et al., 2019; Crimì et al., 2020; Chen et al., 2021). We utilized only pre-treatment CT images due to the limitations of the dataset. We intend to add other image modalities to our data in the future to enhance the performance of the model.

## Conclusion

Tumor shape, size, and heterogeneity, as well as radiation dose to the bladder, may be crucial determinants of pCR. The EBM has the potential to enhance both pCR prediction and model interpretability, which can aid in RT decision-making. Our findings have implications for selecting patients for a “watchful waiting” approach in LARC management.

## Data availability statement

The training dataset that supports the findings of this study is sourced from our institution. The University of Pennsylvania's data-sharing regulations impose restrictions on the availability of these data, which are not accessible to the public. Requests to access the datasets should be directed to the corresponding author at sangho.lee@pennmedicine.upenn.edu.

## Code availability statement

In this study, we only used free and open-source Python packages, which are fully described in Section Materials and methods. An example of Python code that demonstrates how feature extraction in image processing works is archived in the GitHub repository: https://github.com/duwang2015/RDML.

## Ethics statement

The studies involving human participants were reviewed and approved by Institutional Review Board at the University of Pennsylvania. Written informed consent for participation was not required for this study in accordance with the national legislation and the institutional requirements.

## Author contributions

YX, HG, and SL are the study's integrity guarantors. SL, DW, RC, and YX discussed study concepts and design. SL and DW wrote the programs. SL, DW, and YX conducted literature searches and assisted with manuscript preparation. JP and AW guided clinical studies. HG and HZ assisted with data collection and verification. All authors contributed to the article and approved the submitted version.
