# Supplementary material for: Interpretable machine learning for predicting pathologic complete response in patients treated with chemoradiation therapy for rectal adenocarcinoma
Source: Front Artif Intell. 2022 Dec 7;5:1059033. doi: 10.3389/frai.2022.1059033 (PMC9771385; doi:10.3389/frai.2022.1059033)
Supplement: Supplementary file 1 [file Data_Sheet_1.PDF]

Supplementary Material

Radiomics and Dosiomics Features

R and D features used in this study are classified into seven classes: shape (S), first-order (L1), gray level co-occurrence matrix (GLCM), gray level dependence matrix (GLDM), gray level run length matrix (GLRLM), gray level size zone matrix (GLSZM) and neighboring gray tone difference matrix (NGTDM), with GLCM and GLDM belonging to L2 and GLRLM, GLSZM, and NGTDM belonging to L3.

Feature Class

Shape. Three-dimensional size or shape of the region of interest that is independent from the gray level intensity distribution.

Gray level histogram. Histogram-based features that explain the distribution of voxel values inside the mask-defined image region without consideration to spatial relationships.

GLCM. A matrix defined as the distribution of co-occurring pixel values at a given offset over an image. It computes the frequency with which an image contains pairs of pixels with a specified value and offset.

GLDM. A matrix that quantifies the grayscale dependencies present in an image. The number of adjacent voxels that rely on the central voxel from a given distance out is the definition of a gray dependency.

GLRLM. A matrix that measures the gray level runs, which are defined as the number of pixels with the same gray level value.

GLSZM. A matrix that quantifies the gray level zones within an image. The number of connected voxels that have the same gray-level intensity defines a gray-level zone.

NGTDM. A matrix that quantifies the difference between a grayscale value and the average grayscale value of its nearby neighbors.

Feature Statistics

L1: First-order statistics describes the properties of individual pixel values.
L2: Second-order statistics accounts for the spatial inter-dependency or co-occurrence of two pixels at specific relative positions.
L3: Higher-order statistics reveals the spatial relationship among three or more pixels.

Image Processing

To extract R and D features, DICOM data for pretreatment CT scans, RT structure, and RT dose map were loaded. CT images were then cropped onto the bounding box around the GTV with extra padding of 10 voxels. Pre-cropping could speed up extraction and reduce memory requirements, especially for large images with only small regions of interest like GTVs. Padding was applied to all axial, coronal, and sagittal planes, with the size increased in x, y, and z directions by twice the pad distance. Value of padded voxels was set to original gray level intensity, and padding did not exceed original image boundaries.

For R and D, it is most practical to convert RT contours into a binary mask that covers the entire area inside the contours. In this study, GTV contours in the RT structure were used to produce a 3D GTV mask. Only voxels within the GTV retained their original intensities, while all other voxels were converted to zero to ensure that the subsequent R and D calculations were only conducted on the GTV.

Range re-segmentation and intensity outlier filtering were used to remove voxels outside of a specified gray-level range in the GTV which could impact the calculation of R and D features. In this study, we set a fixed threshold at -200 Hounsfield units (HU) and excluded all voxels describing air within the GTV.

Conventional dosimetric factors such as DVH features only utilize partial information contained in the dose distribution. As an extension of R features, D features were extracted to encode the spatial 3D distribution of RT dose, allowing the description of the dose distribution at a high complexity level, distinct from those obtained from DVHs. The integration of D features with the DVH features would constitute a sophisticated tool for evaluating the quality of RT plan by identifying additional dose distribution measures based on D features.

For each patient, the dose distribution (dose grids) within the GTV was resampled with trilinear interpolation to have the same spatial resolution as its corresponding CT, where each voxel held a dose value, to create a 3D dose map. The same R approach was applied to extract D features.

The last image processing step was discretization of image intensities inside the GTV. Voxel intensity data within GTV were discretized using a bin width of 25 HU for CT images and 25 cGy for dose map images. This must be done prior to calculating texture matrices that rely on discrete intensity values. The discretization stage consists of grouping the original values into specific range intervals (bins), which is essentially analogous to creating a histogram. This step is necessary in order to make feature calculation tractable, particularly for the L2 and L3 texture features (1).

Finally, Pyradiomis was used to extract R and D features from the GTV. All R and D features included in this study are listed in Supplementary Table 1.

Supplementary Table 1. R and D features used in this study.

Feature Statistics	Feature Class	Feature Name	No. of Features
	Shape (S)	Elongation, Flatness, LeastAxisLength, MajorAxisLength, Maximum2DDiameterColumn, Maximum2DDiameterRow, Maximum2DDiameterSlice, Maximum3DDiameter, MeshVolume, MinorAxisLength, Sphericity, Sphericity, SurfaceVolumeRatio, VoxelVolume	14
First-order (L1)	Gray level histogram	InterquartileRange, Skewness, Uniformity, Median, Energy, RobustMeanAbsoluteDeviation, MeanAbsoluteDeviation, TotalEnergy, Maximum, RootMeanSquared, 90Percentile, Minimum, Entropy, Range, Variance, 10Percentile, Kurtosis, Mean	18
Second-order (L2)	Gray level co-occurrence matrix (GLCM)	JointAverage, SumAverage, JointEntropy, ClusterShade, MaximumProbability, Idmn, JointEnergy, Contrast, DifferenceEntropy, InverseVariance, DifferenceVariance, Idn, Idm, Correlation, Autocorrelation, SumEntropy, MCC, SumSquares, ClusterProminence, Imc2, Imc1, DifferenceAverage, Id, ClusterTendency	24
	Gray level dependence matrix (GLDM)	GrayLevelVariance, HighGrayLevelEmphasis, DependenceEntropy, DependenceNonUniformity, GrayLevelNonUniformity, SmallDependenceEmphasis, SmallDependenceHighGrayLevelEmphasis, DependenceNonUniformityNormalized, LargeDependenceEmphasis, LargeDependenceLowGrayLevelEmphasis, DependenceVariance, LargeDependenceHighGrayLevelEmphasis, SmallDependenceLowGrayLevelEmphasis, LowGrayLevelEmphasis	14
Higher-order (L3)	Gray level run length matrix (GLRLM)	ShortRunLowGrayLevelEmphasis, GrayLevelVariance, LowGrayLevelRunEmphasis, GrayLevelNonUniformityNormalized, RunVariance, GrayLevelNonUniformity, LongRunEmphasis, ShortRunHighGrayLevelEmphasis, RunLengthNonUniformity, ShortRunEmphasis, LongRunHighGrayLevelEmphasis, RunPercentage, LongRunLowGrayLevelEmphasis, RunEntropy, HighGrayLevelRunEmphasis, RunLengthNonUniformityNormalized	16
	Gray level size zone matrix (GLSZM)	GrayLevelVariance, SmallAreaHighGrayLevelEmphasis, GrayLevelNonUniformityNormalized, SizeZoneNonUniformityNormalized, SizeZoneNonUniformity, GrayLevelNonUniformity, LargeAreaEmphasis, ZoneVariance, ZonePercentage, LargeAreaLowGrayLevelEmphasis, LargeAreaHighGrayLevelEmphasis, HighGrayLevelZoneEmphasis, SmallAreaEmphasis, LowGrayLevelZoneEmphasis, ZoneEntropy, SmallAreaLowGrayLevelEmphasis	16
	Neighboring gray tone difference matrix (NGTDM)	Coarseness, Complexity, Strength, Contrast, Busyness	5

Supplementary Table 2. Performances of EBM models in pCR prediction with top ten multi-view input feature sets and the single-view input feature set are compared. For each input, the training AUC, testing AUC, and selected features are presented.

Input Feature Sets			Selected Features	Performance	
				AUC	
CP+DVH	Radiomics	Dosiomics		Training	Testing
√	S+L1+L2	–	Bladder_Dmax, GTV_R_Variance, GTV_Elongation, GTV_LeastAxisLength, GTV_Maximum2DDiameterColumn	0.762	0.820
√	S+L1	L1	GTV_R_Variance, GTV_Elongation, GTV_Maximum2DDiameterColumn, GTV_D_90Percentile	0.762	0.809
√	S+L1+L2	L1	GTV_R_Variance, GTV_Elongation, GTV_Maximum2DDiameterColumn, GTV_D_90Percentile	0.762	0.809
√	S+L1	L2	GTV_Elongation, GTV_LeastAxisLength, GTV_Maximum2DDiameterColumn, GTV_D_glcm_DifferenceAverage	0.764	0.699
√	S+L1	L1+L2	GTV_Elongation, GTV_LeastAxisLength, GTV_Maximum2DDiameterColumn, GTV_D_glcm_DifferenceAverage	0.770	0.694
√	S+L1+L2	L1+L3	GTV_R_Variance, GTV_Elongation, GTV_LeastAxisLength, GTV_Maximum2DDiameterColumn, GTV_Maximum2DDiameterSlice, GTV_D_90Percentile, GTV_D_glrlm_LongRunEmphasis, GTV_D_ngtdm_Complexity	0.781	0.694
√	S+L1	L2+L3	GTV_R_Variance, GTV_Elongation, GTV_LeastAxisLength, GTV_MajorAxisLength, GTV_Maximum2DDiameterColumn, GTV_Maximum2DDiameterSlice, GTV_D_glszm_SizeZoneNonUniformity, GTV_D_ngtdm_Complexity	0.769	0.694
√	S+L1	L1+L3	GTV_Variance, GTV_Elongation, GTV_LeastAxisLength, GTV_Maximum2DDiameterColumn, GTV_D_90Percentile, GTV_D_glrlm_LongRunEmphasis, GTV_D_glszm_SizeZoneNonUniformity, D_ngtdm_Complexity	0.782	0.688
√	S+L1+L2+L3	L2	GTV_R_Variance, GTV_Elongation, GTV_LeastAxisLength, GTV_Maximum2DDiameterColumn, GTV_Maximum2DDiameterSlice, GTV_R_glszm_GrayLevelNonUniformity, GTV_D_glcm_Correlation	0.740	0.688
√	L2+L3	L2	GTV_R_gldm_SmallDependenceLowGrayLevelEmphasis	0.638	0.682
√	S+L1+L2+L3	L1+L2+L3	GTV_R_Variance<br>GTV_Elongation<br>GTV_LeastAxisLength<br>GTV_Maximum2DDiameterColumn<br>GTV_R_gldm_SmallDependenceLowGrayLevelEmphasis<br>GTV_R_glszm_GrayLevelNonUniformity<br>GTV_D_glrlm_LongRunEmphasis<br>GTV_D_glszm_SizeZoneNonUniformity<br>GTV_D_ngtdm_Complexity	0.772	0.624

Supplementary Table 3. Performances of XGB models in pCR prediction with top ten multi-view input feature sets and the single-view input feature set are compared. For each input, the training AUC, testing AUC, and selected features are presented.

**Input Feature Sets**			**Selected Features**	**Performance**	
				AUC	
CP+DVH	Radiomics	Dosiomics		Training	Testing
√	S+L1+L2	–	Bladder_Dmax, GTV_R_Variance, GTV_Elongation, GTV_LeastAxisLength, GTV_Maximum2DDiameterColumn	0.820	0.828
√	S+L1	L1	GTV_R_Variance, GTV_Elongation, GTV_Maximum2DDiameterColumn, GTV_D_90Percentile	0.844	0.823
√	S+L1+L2	L1	GTV_R_Variance, GTV_Elongation, GTV_Maximum2DDiameterColumn, GTV_D_90Percentile	0.844	0.823
√	S+L1+L2+L3	L2	GTV_R_Variance, GTV_Elongation, GTV_LeastAxisLength, GTV_Maximum2DDiameterColumn, GTV_Maximum2DDiameterSlice, GTV_R_glszm_GrayLevelNonUniformity, GTV_D_glcm_Correlation	0.829	0.688
√	S+L1	L2+L3	GTV_R_Variance, GTV_Elongation, GTV_LeastAxisLength, GTV_MajorAxisLength, GTV_Maximum2DDiameterColumn, GTV_Maximum2DDiameterSlice, GTV_D_glszm_SizeZoneNonUniformity, GTV_D_ngtdm_Complexity	0.868	0.672
√	–	L1	GTV_D_90Percentile	0.690	0.656
√	L2	L1	GTV_D_90Percentile	0.690	0.656
√	–	L1+L2	GTV_D_90Percentile	0.690	0.656
√	L2	L1+L2	GTV_D_90Percentile	0.690	0.656
√	L2	L1+L2+L3	GTV_D_90Percentile, GTV_D_glszm_SizeZoneNonUniformity, GTV_D_ngtdm_Complexity	0.780	0.651
√	S+L1+L2+L3	L1+L2+L3	GTV_R_Variance, GTV_Elongation, GTV_LeastAxisLength, GTV_Maximum2DDiameterColumn, GTV_R_gldm_SmallDependenceLowGrayLevelEmphasis, GTV_R_glszm_GrayLevelNonUniformity, GTV_D_glrlm_LongRunEmphasis, GTV_D_glszm_SizeZoneNonUniformity, GTV_D_ngtdm_Complexity	0.814	0.484

Supplementary Table 4. Performances of RF models in pCR prediction with top ten multi-view input feature sets and the single-view input feature set are compared. For each input, the training AUC, testing AUC, and selected features are presented.

Input Feature Sets			Selected Features	Performance	
				AUC	
CP+DVH	Radiomics	Dosiomics		Training	Testing
√	S+L1+L2	–	Bladder_Dmax, GTV_R_Variance, GTV_Elongation, GTV_LeastAxisLength, GTV_Maximum2DDiameterColumn	0.821	0.828
√	S+L1	L1	GTV_R_Variance, GTV_Elongation, GTV_Maximum2DDiameterColumn, GTV_D_90Percentile	0.854	0.774
√	S+L1+L2	L1	GTV_R_Variance, GTV_Elongation, GTV_Maximum2DDiameterColumn, GTV_D_90Percentile	0.854	0.774
√	S+L2+L3	L2	GTV_R_gldm_SmallDependenceLowGrayLevelEmphasis	0.705	0.731
√	S+L1	–	GTV_Elongation, GTV_LeastAxisLength, GTV_Maximum2DDiameterColumn	0.772	0.715
√	S+L1	L2	GTV_Elongation, GTV_LeastAxisLength, GTV_Maximum2DDiameterColumn, GTV_D_glcm_DifferenceAverage	0.834	0.683
√	S+L1+L2	L1+L3	GTV_R_Variance, GTV_Elongation, GTV_LeastAxisLength, GTV_Maximum2DDiameterColumn, GTV_Maximum2DDiameterSlice, GTV_D_90Percentile, GTV_D_glrlm_LongRunEmphasis, GTV_D_ngtdm_Complexity	0.902	0.661
√	S+L1+L2+L3	L2	GTV_R_Variance, GTV_Elongation, GTV_LeastAxisLength, GTV_Maximum2DDiameterColumn, GTV_Maximum2DDiameterSlice, GTV_R_glszm_GrayLevelNonUniformity, GTV_D_glcm_Correlation	0.841	0.656
√	S+L1+L2	L2	GTV_Elongation, GTV_LeastAxisLength, GTV_Maximum2DDiameterColumn, GTV_D_glcm_DifferenceEntropy	0.817	0.645
√	S+L1+L3	–	GTV_Elongation, GTV_MajorAxisLength, GTV_Maximum2DDiameterColumn, GTV_R_glszm_GrayLevelNonUniformity	0.812	0.634
√	S+L1+L2+L3	L1+L2+L3	GTV_R_Variance, GTV_Elongation, GTV_LeastAxisLength, GTV_Maximum2DDiameterColumn, GTV_R_gldm_SmallDependenceLowGrayLevelEmphasis, GTV_R_glszm_GrayLevelNonUniformity, GTV_D_glrlm_LongRunEmphasis, GTV_D_glszm_SizeZoneNonUniformity, GTV_D_ngtdm_Complexity	0.858	0.575

Supplementary Table 5. Performances of SVM models in pCR prediction with top ten multi-view input feature sets and the single-view input feature set are compared. For each input, the training AUC, testing AUC, and selected features are presented.

**Input Feature Sets**			**Selected Features**	**Performance**	
				AUC	
CP+DVH	Radiomics	Dosiomics		Training	Testing
√	S+L1+L2	–	Bladder_Dmax, GTV_R_Variance, GTV_Elongation, GTV_LeastAxisLength, GTV_Maximum2DDiameterColumn	0.725	0.774
√	S+L1+L2+L3	L2	GTV_R_Variance, GTV_Elongation, GTV_LeastAxisLength, GTV_Maximum2DDiameterColumn, GTV_Maximum2DDiameterSlice, GTV_R_glszm_GrayLevelNonUniformity, GTV_D_glcm_Correlation	0.770	0.774
√	S+L1	L1	GTV_R_Variance, GTV_Elongation, GTV_Maximum2DDiameterColumn, GTV_D_90Percentile	0.782	0.747
√	S+L1+L2	L1	GTV_R_Variance, GTV_Elongation, GTV_Maximum2DDiameterColumn, GTV_D_90Percentile	0.782	0.747
√	S+L1	L1+L3	GTV_R_Variance, GTV_Elongation, GTV_LeastAxisLength, GTV_Maximum2DDiameterColumn, GTV_D_90Percentile, GTV_D_glrlm_LongRunEmphasis, GTV_D_glszm_SizeZoneNonUniformity, GTV_D_ngtdm_Complexity	0.782	0.720
√	S+L1	L2+L3	GTV_R_Variance, GTV_Elongation, GTV_LeastAxisLength, GTV_MajorAxisLength, GTV_Maximum2DDiameterColumn, GTV_Maximum2DDiameterSlice, GTV_D_glszm_SizeZoneNonUniformity, GTV_D_ngtdm_Complexity	0.743	0.694
√	S+L1+L2	L2	GTV_Elongation, GTV_LeastAxisLength, GTV_Maximum2DDiameterColumn, GTV_D_glcm_DifferenceEntropy	0.839	0.672
√	S+L1	L2	GTV_Elongation, GTV_LeastAxisLength, GTV_Maximum2DDiameterColumn, GTV_D_glcm_DifferenceAverage	0.791	0.656
√	S+L1	–	GTV_Elongation, GTV_LeastAxisLength, GTV_Maximum2DDiameterColumn	0.613	0.645
√	L2+L3	L2	GTV_R_gldm_SmallDependenceLowGrayLevelEmphasis	0.750	0.640
√	S+L1+L2+L3	L1+L2+L3	GTV_R_Variance, GTV_Elongation, GTV_LeastAxisLength, GTV_Maximum2DDiameterColumn, GTV_R_gldm_SmallDependenceLowGrayLevelEmphasis, GTV_R_glszm_GrayLevelNonUniformity, GTV_D_glrlm_LongRunEmphasis, GTV_D_glszm_SizeZoneNonUniformity, GTV_D_ngtdm_Complexity	0.795	0.522

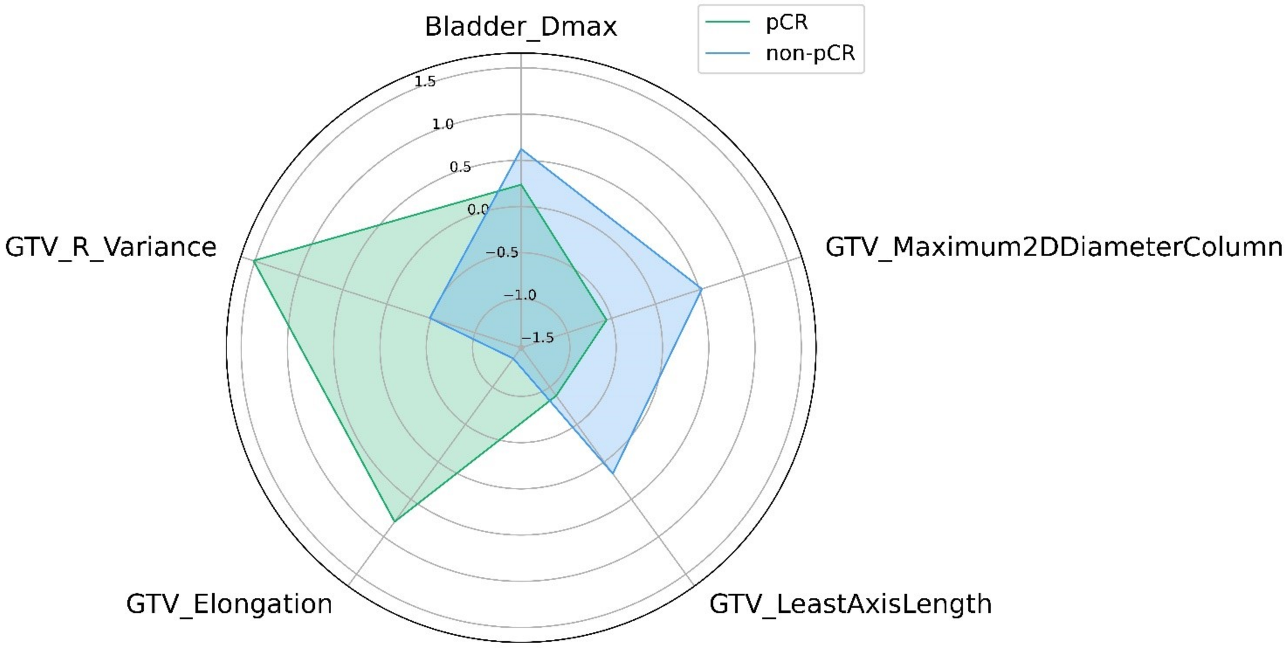

Supplementary Figure 1. Five selected features including bladder_Dmax, GTV_R_Variance, GTV_Elongation, GTV_LeastAxisLength, and GTV_Maximum2DDiameterColumn are compared between a pCR patient and a non-pCR patient chosen from the test dataset. For unbiased visual comparison, feature values are standardized using Z scores so that all feature values are centered around mean with a unit standard deviation. The patient who achieved pCR had a higher variance in CT intensities and a more circular tumor shape, whereas the patient who did not reach pCR had a higher maximum dose to the bladder and a larger tumor size.

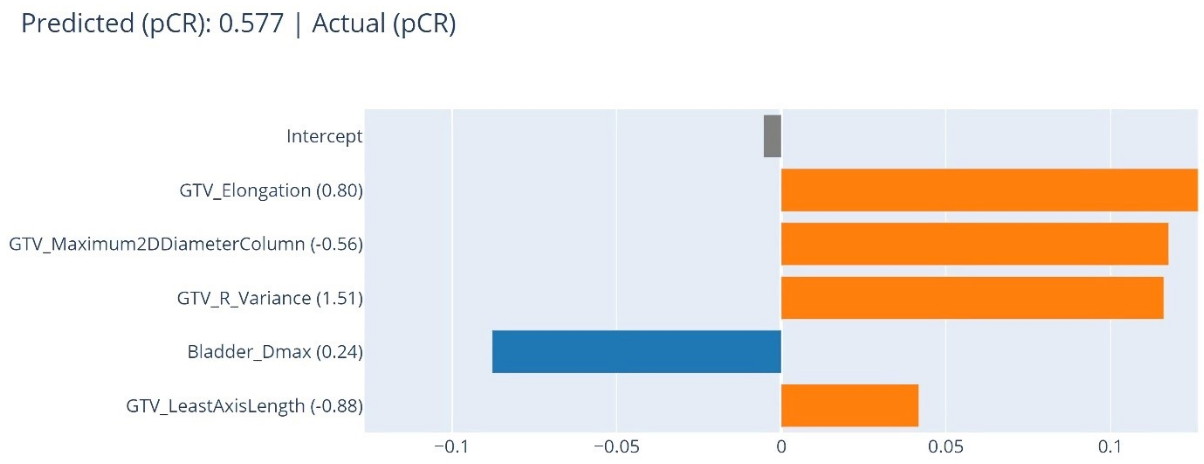

(a)

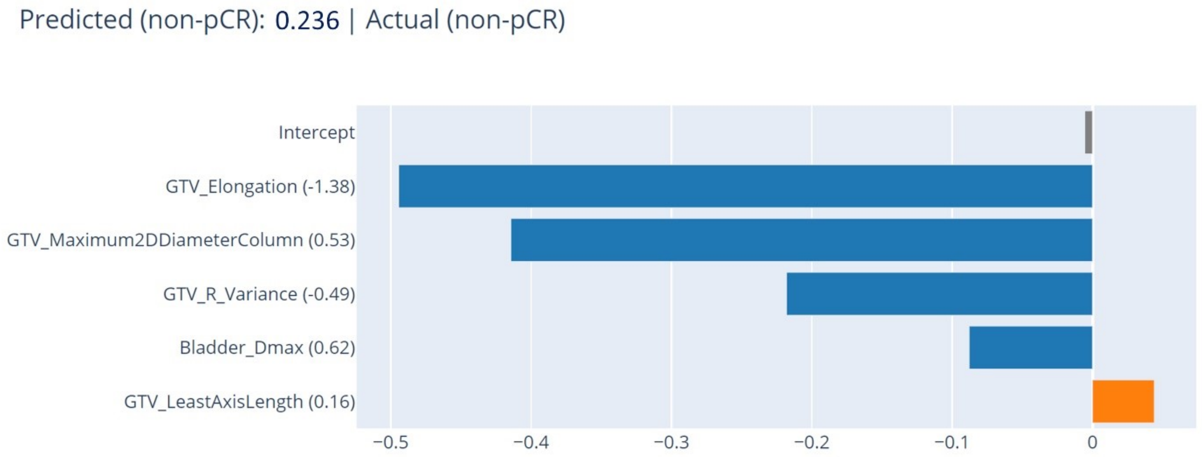

(b)

Supplementary Figure 2. Comparison of local explanations of the EBM model between (a) a patient who achieved pCR and (b) a patient who did not achieve pCR. The y-axis indicates the feature value (standardized), while the x-axis represents the value of each feature's smooth function on a single observation. A positive smooth function value indicates that the feature promotes achieving pCR, whereas a negative smooth function value suggests that the feature does not. The probability of pCR predicted by the EBM model is shown in the title.

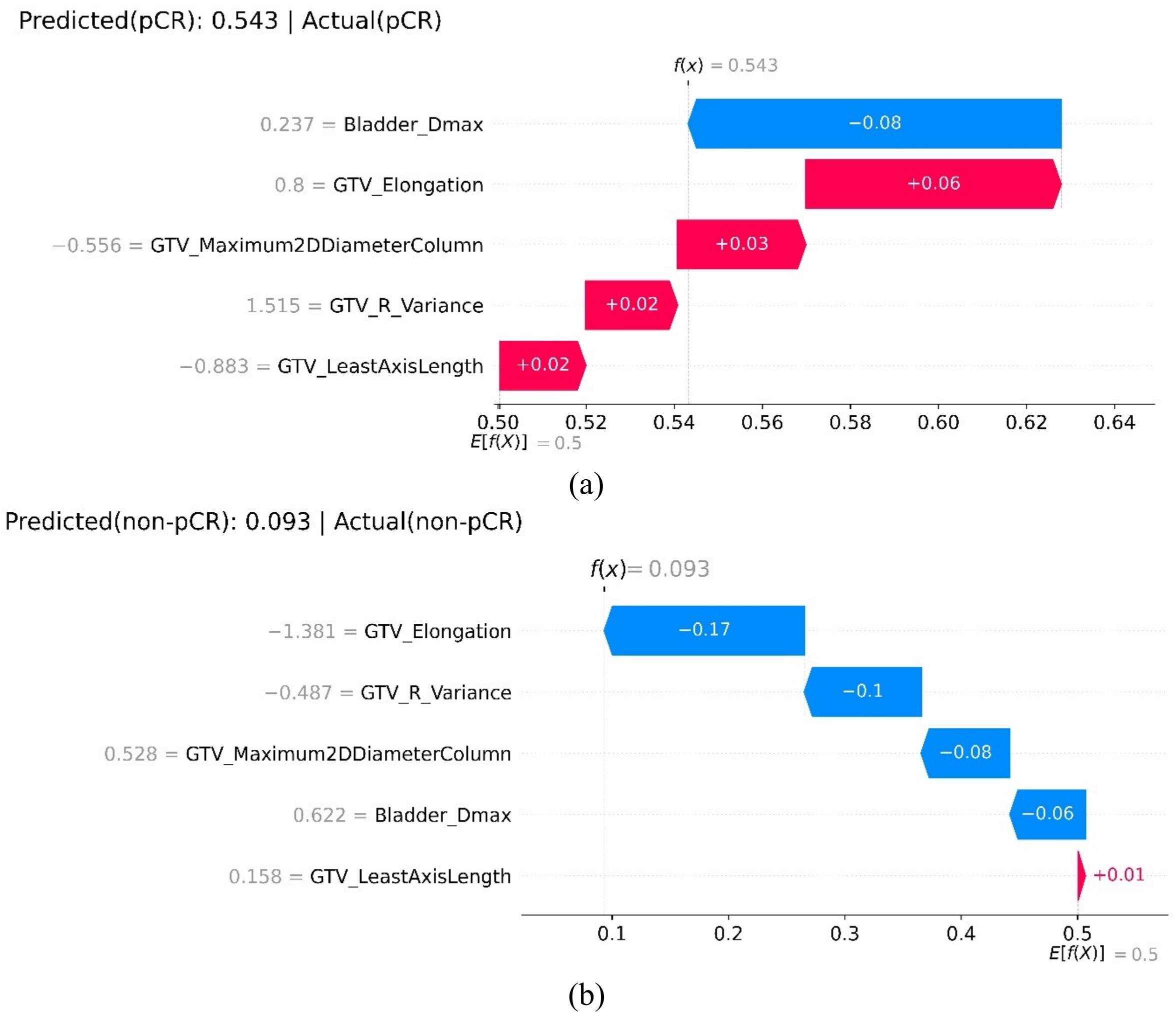

Supplementary Figure 3. Comparison of local explanations of the RF model between (a) a patient who achieved pCR and (b) a patient who did not achieve pCR. The y-axis indicates the feature value (standardized), while the x-axis represents each feature's impact on the prediction of a single observation. The E[f(x)] represents the SHAP base value which is the average of the model output over the training dataset and f(x) is the model's prediction for the current case. Features are ranked based on the magnitude of their impact with red bar representing the positive contribution and the blue bar representing the negative contribution. The probability of pCR predicted by the EBM model is shown in the title.

Reference

(1) van Timmeren JE, Cester D, Tanadini-Lang S, Alkadhi H, Baessler B. Radiomics in medical imaging—"how-to" guide and critical reflection. Insights Imaging 2020 -8-12;11.
